# Sitagliptin pretreatment in diabetes patients presenting with acute coronary syndrome: results from the Acute Coronary Syndrome Israeli Survey (ACSIS)

**DOI:** 10.1186/1475-2840-12-53

**Published:** 2013-03-28

**Authors:** Eyal Leibovitz, Shmuel Gottlieb, Ilan Goldenberg, Natalie Gevrielov-Yusim, Shlomi Matetzky, Dov Gavish

**Affiliations:** 1 Department of Internal Medicine “A” Wolfson Medical Center, Holon, Israel; 2 Neufled Cardiac Research Institute, Sheba Medical Center, Ramat Gan, Israel; 3 Heart Institute, Bikur Cholim Campus, Cardiology Department, Shaare Zedek Medical center, Jerusalem, Israel; 4 Sackler Medical School, Tel Aviv University, Tel Aviv, Israel

**Keywords:** DPP4 inhibitors, Sitagliptin, Acute coronary syndrome, Diabetes mellitus

## Abstract

**Background:**

Chronic treatment with currently available oral hypoglyemic medications may result in a differential effect on the clinical presentation of diabetic patients with acute coronary syndrome (ACS).

**Methods:**

We evaluated presentation characteristics and the risk for in-hospital complications and 30-day major adverse cardiovascular events (MACE) among 445 patients with diabetes mellitus enrolled in the Acute Coronary Syndrome Israeli Survey (ACSIS) 2010. Patients were categorized into 3 groups according to glucose lowering medications at time of admission for ACS: 1) DPP 4 inhibitors (as monotherapy or in combination; DPP4i), 2) Metformin (monotherapy or in combination, excluding DPP4i) and 3) other oral hypoglycemics.

**Results:**

Patients in the DPP4i group displayed similar baseline clinical characteristics to the other 2 groups, with the exception of a younger age and a lower frequency of prior coronary heart disease and chronic renal failure. Medical therapy with DPP4i was associated with a significantly lower in-hospital complication rate (post MI angina, re-infarction, pulmonary edema, infections, acute renal failure and better KILLIP class) (9.7%), lower rates of 30-day MACE (12.9%) and a shorter hospital stay (5.4 ± 3.8 days) as compared with patients treated with metformin (24.4%, 31.6% and 5.6 ± 5.0 days respectively) or other oral hypoglycemic drugs (45.5%, 48.5% and 7.5 ± 6.5 days respectively). Consistently, multivariate logistic regression modeling revealed that treatment with DPP4i was associated with a lower risk for in-hospital complications (OR = 0.129, p = 0.002) and 30-day MACE (OR = 0.157, p = 0.002) compared with other oral hypoglycaemic therapy.

**Conclusions:**

Our data suggests that chronic treatment with DPP4i may have cardioprotective effects in diabetes patients presenting with acute coronary syndrome.

## Introduction

The prevalence of diabetes is increasing worldwide, and it is estimated that 7.7% or 439 million of the global adult population will have diabetes by 2030 [1]. The aim of diabetes treatment is to reduce glucose levels and to achieve the recommended HbA1C level of <7%, in order to reduce the occurrence of vascular complications [2]. Controlling glucose levels is difficult and requires usage of glucose lowering medications in most if not all cases. This demand for anti-diabetes medications has driven the pharmaceutical industry to develop new drugs.

Several new anti-diabetes agents were approved for treatment due to their glucose lowering effect, but without proof of reduction of hard end-points, such as major adverse cardiovascular events (MACE). One of the drugs approved for diabetes control was Rosiglitazone, a PPAR gamma agonist that was shown to significantly improve glucose levels among patients with diabetes [3]. This drug was recently removed from the market following FDA warnings for possible cardiovascular complications [4]. Two other drug families that were recently approved for treatment of diabetes mellitus are the Glucagone like Peptide-1 (GLP-1) analogues and Di Peptidyl Peptidase IV (DPP4) inhibitors, both act via the incretin pathway. Glucagone like Peptide-1 enhances the secretion of insulin from pancreatic beta cells following meals, as well as prevent apoptosis of these cells via sub-cellular pathways such as JNK [5]. These peptides have a short half-life, and are metabolized by the enzyme DPP-4 to their inactive form.

The GLP-1 analogues are peptides that are resistant to the activity of DPP-4, and therefore have a longer half-life and enhanced activity. DPP-4 inhibitors (DPP4i) increase the half-life of the incretins by preventing their degradation. Both drug families have been shown to have beneficial effects on blood glucose and enhance the control rate of diabetes [6,7]. Recent data suggest that DPP4i may have antithrombotic and anitinflmmatory effects [8], and thus may be protective in patients who present with acute coronary syndrome (ACS). However, there are limited data regarding the effects of different classes oral hypoglycemic medications on the clinical presentation of ACS.

Accordingly, the purpose of this study was to examine the effects of glucose lowering medications on the type and severity of myocardial damage, in-hospital complications and 30-day MACE among diabetes patients admitted to the hospital with acute coronary syndrome.

## Methods

The parameters in this study were derived from the Acute Coronary Syndrome Israeli Survey (ACSIS) 2010, a bi-annual nationwide survey of acute coronary syndrome patients that were admitted to all 26 public hospitals in Israel during a 2 month period of March-April 2010. Methods on data acquisition are specified elsewhere [9].

Included in this sub-analysis were all patients diagnosed with type 2 diabetes mellitus that received oral hypoglycemic medications. Excluded were patients who were on chronic insulin treatment (all types either as monotherapy or in combination with oral hypoglycemic medications) and those newly diagnosed with diabetes mellitus during the admission associated with the coronary event. Duration of treatment with oral hypoglycemic medication prior to hospitalization was not recorded.

The ACSIS survey is a nationwide survey conducted in several hospitals across Israel. The study was approved by each of the local IRB’s as an observational study, precluding the need for informed consent.

### Definitions and endpoints

Patients were divided into 3 groups according to the type of oral hypoglycemic medications received: 1) patients receiving DPP4i either alone or in combination with any other oral hypoglycemic medications, 2) patients receiving metformin either as monotherapy or in combination with other oral hypoglycemic medications (excluding DPP4i), and 3) other oral hypoglycemic medications, excluding DPP4i and Metformin.

Composite endpoints included: in-hospital complication (one or more of either acute renal failure, pulmonary edema, infection, killip ≥ 2 on admission) [10] and 30-day MACE (one or more of either stent thrombosis, urgent revascularization, post event ischemia, 30 day mortality, re-infarction or re-ischemia, re-admission and stroke/TIA), In-hospital and 30-day outcome data were ascertained by hospital chart review, telephone contact, and clinical follow-up data. Mortality data during hospitalization and at 30 days were determined for all patients from hospital charts and by matching identification numbers of patients with the Israeli National Population Register.

### Statistical analysis

Baseline characteristics including risk factors, clinical characteristic, laboratory values, hospital and 30-day course of the patients were compared by the prespecified three oral hypoglycemic treatment groups. Comparison of categorical variables was performed with Chi-square analysis and comparison of continuous variables was performed with the nonparametric Wilcoxon rank-sum and Kruskal-Wallis tests as appropriate. A logistic regression model was used to evaulate the effect of diabetes treatment on the in-hospital and 30-day composite outcome measures. Prespecified covariates in the multivariate models included the baseline diabetes treatment groups (using the other oral treatment [group 3] as the referemce group), age, gender, history of CHF, history of renal failure, history of peripheral vascular disease, history of hypertension, history of dyslipidemia and history of ischemic heart disease. Odds ratios with 95% confidence intervals were calculated using other oral treatment (group 3) as the referemce group.

The statistical software used for the analyses was SAS version 9.2 (SAS institute, Cary, North Carolina). A two-sided p-value <0.05 was used for declaring statistical significance.

## Results

Of the 2193 patients enrolled in the 2010 ACSIS survey, 877 patients had diabetes (39.9%) on admission. Of the patients with diabetes on admission, 432 were excluded from the analysis because: 1) data on hypoglycemic treatment was missing from 217 cases, 2) 199 were treated with insulin prior to admission (either as monotherapy or in combination with oral hypoglycemic medications) and 3) 13 patients had diabetes mellitus type 1. Four hundred and forty five diabetes patients fulfilled the inclusion and exclusion criteria and comprised the present study population: representing 20.3% of the entire database and 50.7% of the diabetes sub-population. The mean age of the patients included in this analysis was 67.8 years and 320 (71.9%) were males.

### Clinical presentation and in-hospital management by oral hypoglycemic treatment group

Of the 445 patients, 31 patients (7%) were included in the DPP4 inhibitors group (DPP4i), 348 patients (78%) in the metformin group and 66 patients (15%) received other hypoglycemic medications, and were included in the “other oral” group. Patients in the “other oral” group were older with higher rates of chronic renal failure and PVD (Table 1). No significant difference was noted between the DPP4i and the Metformin groups. In our dataset, Sitagliptin was the only DPP4 inhibitor prescribed to the patients, either as monotherapy, or in combination with other medications. There was no significant difference in blood pressure values and LDL-cholsterol levels; However, patients on Metformin tended to have a lower fasting plasma glucose on admission, whereas patients in the “other oral” group had a higher creatinine level, associated with increased rate of chronic renal failure. In addition, treatment with DPP4 inhibitors was associated with a lower admission KILLIP class, as compared to the “metformin” and “other oral” groups (Table 1). There were no statistically significant differences in the rate and type of coronary reprerfusion treatment for ST-elevation ACS (26.3% of patient population) across the three treatment groups, nor were there any differences noted in the in-hospital medication and intervention treatment (Table 2).

**Table 1:** Demographic parameters and co-morbidities according to treatment group

		**DPP4i**	**Metformin**	**Other oral**	**p Value**
		**n = 31**	**n = 348**	**n = 66**	
**Demographics**					
	Age (years)	64.3 ± 9.7	67.3 ± 11.3	72.2 ± 12.9	0.001
	Male sex (%)	84	71	70	0.30
*	BMI (kg/m^2^)	30.1 ± 4.4	28.4 ± 4.7	28.3 ± 3.6	0.04
**Co-morbidities**					
†	Prior MI (%)	41.9	37.7	48.5	0.25
‡	Prior PCI (%)	51.6	43.2	37.9	0.44
§	Prior CHF (%)	9.7	15.2	25.8	0.06
||	Prior CRF (%)	22.6	13.2	43.9	<0.001
¶	Prior PVD (%)	16.1	10.7	25.0	0.007
	Prior stroke (%)	6.5	12.4	18.2	0.24
	Hypertension (%)	83.9	81.8	90.9	0.20
	Dyslipidemia (%)	87.1	88.1	80.0	0.21
	Smoker (%)	20.0	26.2	19.7	0.45
**Admission laboratory parameters and values**					
^*^	Admission SBP (mmHg)	150 ± 28	144 ± 29	144 ± 32	0.68
†	Admission DBP (mmHg)	83 ± 16	81 ± 17	77 ± 16	0.13
	Admission heart rate (bpm)	80 ± 20	82 ± 19	85 ± 22	0.41
‡	Maximal CK (IU/L)	121.5 (23–1994)	167.0 (11–1994)	218.0 (41–1808)	0.20
	Glucose (mg/dL)	195 (111–429)	159 (71–792)	189 (40–506)	0.09
	Leukocytes (cmm^3^x1000)	10.4 ± 3.7	10.7 ± 4.3	10.9 ± 4.2	0.82
§	LDL-c (mg/dL)	86 ± 27	90 ± 34	84 ± 30	0.58
	Creatinine (mg/dL)	1.0 (0.5–2.3)	1.0 (0.5–7.6)	1.4 (0.7–6.5)	<0.001
	Admission KILLIP >1 (%)	9.7	16.4	34.8	<0.001

*BMI – Body Mass Index.

§CHF – Congestive Heart Failure.

||CRF – Chronic Renal Failure.

†MI – Myocardial infarction.

‡PCI – Percutaneous Coronary Intervention.

¶PVD – Peripheral Vascular Disease.

**Table 2:** In-hospital management according to treatment groups

		**DPP4i**	**Metformin**	**Other Oral**	**p Value**
		**n = 31**	**n = 348**	**n = 66**	
	Aspirin (%)	96.8	97.1	97.0	0.99
	Clopidogrel (%)	96.8	92.2	90.9	0.59
*	LMW Heparin (%)	48.4	52.6	48.5	0.77
†	UF Heparin (%)	46.7	37.1	36.4	0.57
‡	ACE Inhibitors (%)	76.7	72.1	60.6	0.13
§	ARB (%)	10.0	14.1	9.1	0.48
	Beta Blockers (%)	87.1	82.8	77.3	0.43
	Statins	93.5	96.0	95.5	0.81
Coronary interventions during admission					
	STEMI reperfusion (%)	71	66	52	0.06
||	PCI (%)	70.4	72.0	58.3	0.24
	Coronary stenting (%)	88.9	88.7	81.0	0.58
	Coronary bypass Surgery (%)	11.1	5.6	13.9	0.12

‡ACEIs – Angiotensin Converting Enzyme.

§Angiotensin Receptor Blockers.

*LMW – Low Molecular Weight.

||PCI – Percutaneous Coronary.

Intervention.

†UF – Unfractionated

### In-hospital and 30-day outcomes by oral hypoglycemic treatment group

**Figure 1:**
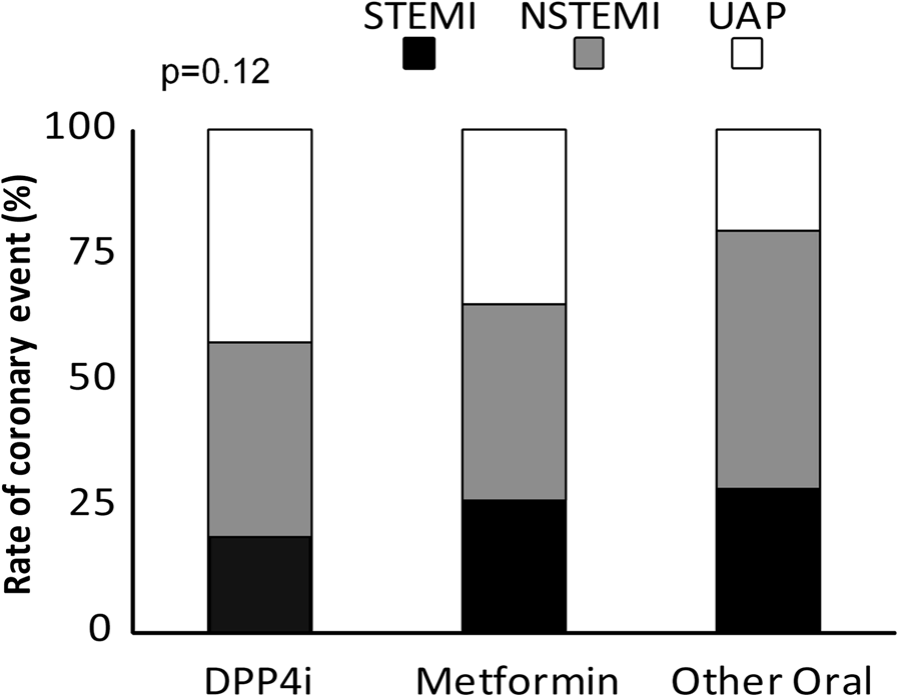
Acute coronary event type by treatment group.

Type of coronary event varied: 26.3% of the patients were diagnosed with a STEMI, 40.9% with a non-STEMI and 32.8% with UAP. There was no difference in the prevalence of the type of coronary event through the treatment groups (Figure 1).

**Figure 2:**
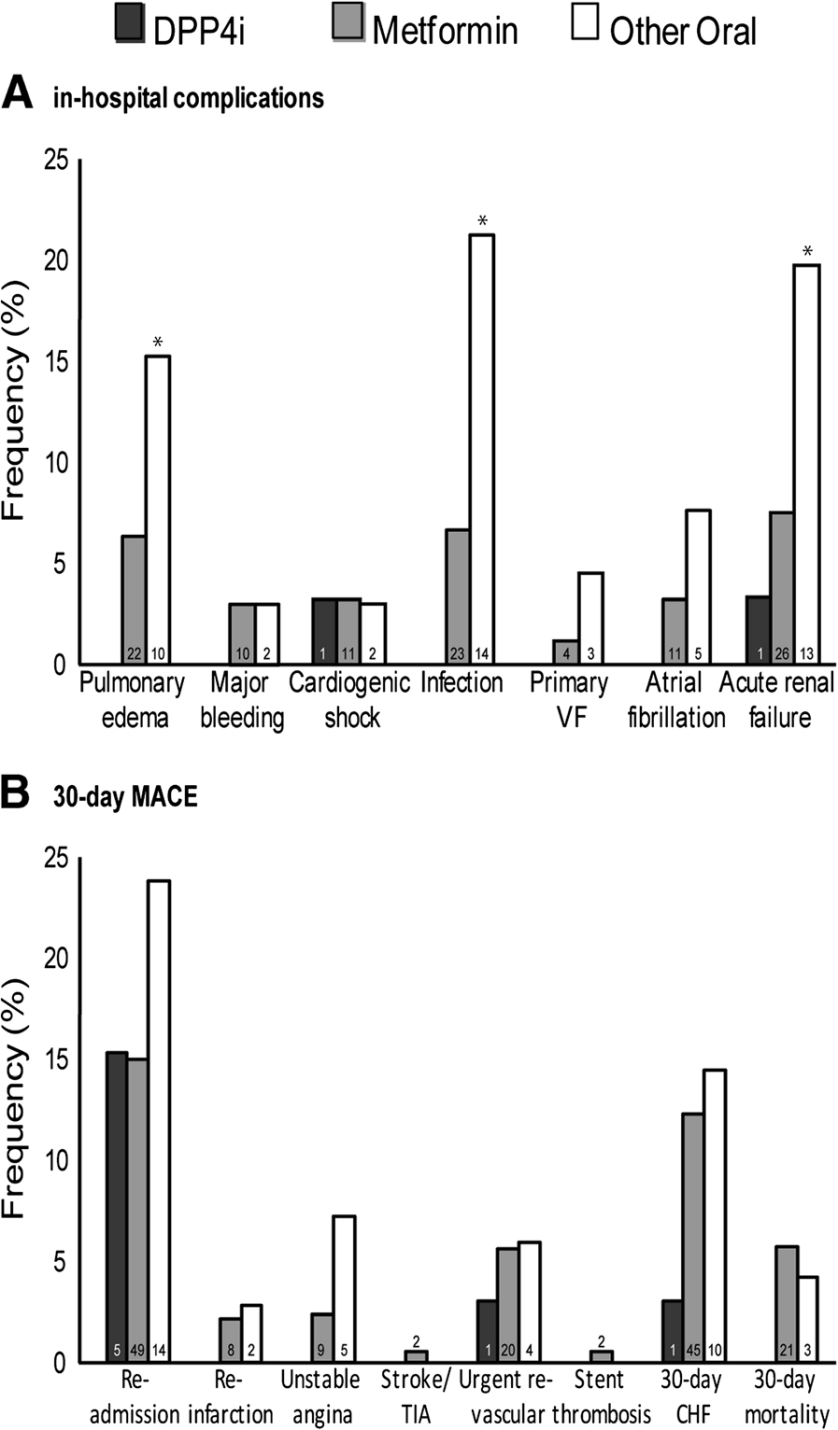
**Rate of in-hospital complications (panel A) and 30-day major cardiovascular events (panel B) by treatment group (Number of events appear at the base of each specific column).** For panel **A**, composite endpoint (compared to other oral) were: 0.129 (95% CI 0.036–0.465) for the DPP4i group and 0.388 (95% CI 0.225–0.667) for the Metformin group. For panel **B**, composite endpoint (compared to other oral) were: 0.157 (95% CI 0.05–0.50) for the DPP4i group and 0.491 (95% CI 0.288–0.837) for the Metformin group. “*” indicates a p value of less than 0.05.

The rate and number of in hospital complications and 30-day major cardiovascular events are specified in Figure 2. Rates of pulmonary edema, acute renal failure and infections during hospitalizations were higher in the other oral group as compared to the DPP4i and metformin groups (p = 0.01, 0.003, 0.0009 respectivly). When comparing specific 30-day MACE events, the occurrence of unstable AP tended to be higher in the other oral group (p = 0.06) compared to the DPP4i group. In addition, length of stay varied across treatment groups. Median length of stay was 5 days (3–7 days for Q1-Q3) in the DPP4i group, 4 days [3-7] in the Metformin group and 6 days [3-10] in the Other Oral group (p = 0.02).

When analysing the composite of in-hospital complications, type of diabetes treatment was a significant contributor to the complication rate. Multivariate logistic regression analysis (see Methods) revealed that treatment with DPP4i (OR = 0.21, 0.05–0.87) were independently associated with a lower risk for in-hospital complications. Other variables associated with in-hospital complications were: prior CHF (OR = 3.2, 95% confidence interval 1.72–5.95) and age (1 year increment) (OR = 1.08, 1.06–1.11) (c for the model 0.804). The risk for the composite 30-day MACE endpoint was also significantly influenced by diabetes treatment. Multivariate logistic regression models (see Methods) revealed that DPP4i treatment was independently associated with lower composite 30-day MACE (OR = 0.27, 0.08–0.96). Other parameters associated with increased 30-day MACE were: CHF (OR = 3.02, 1.64–5.57) and age (OR = 1.07, 1.04–1.09) (c for the model =0.769).

## Discussion

Despite the beneficial effect of DPP4i on glucose levels [11], the high number of substrates of the DPP-4 [12] raise the question whether one substrate (or more) would have negative effects to undermine the beneficial effects of glucose lowering in diabetes patients. Such an effect was documented by Jackson et al. [13,14], who showed that treatment with Sitagliptin increased the renovascular effects of angiotensin II. This was attributed to enhanced effect of peptide YY_1-36_, one of the substrate of the DPP-4 enzyme. The clinical singnificance of this finding is unknown, however, tbe majority of the substrates are peptides with little or no cardiovascular effect [12].

The main effect of DPP4i is associated with GLP-1. Altough there are no randomized clinical studies on the effect of GLP-1 on cadiovascular disease, treatment with GLP-1 analogues may be associated with improved status of cardiovascular risk factors such as hypertension and body weight [15-17]. Besides the effects on CVD risk factors, GLP-1 was found to have a beneficial effect on infarct size in animal models [18], as well as among diabetes patients undergoing CABG surgery, where the untreated group required inotropic and vasoactive infusions more frequently compared to those treated with GLP-1 [19]. Altough the number of studies evaluating the effects of DPP4i on CVD are limited, a beneficial effect of DPP4i treatment on blood pressure was observed in a study of non diabetic population [20]. However, the magnitude of blood pressure reduction was minimal. Additional effects on atherosclerosis were found in a model of LDL receptor knockout mice, as well as in APOE knockout model, where DPP4i was associated with less atherosclerosis. The effect is attributed to reduced monocyte assocaited inflammation [21] and augmented effect of GLP-1 in macrophages and endothelial cells [22].

Besides the effect of DPP4i on CVD risk factors and atherosclerosis, an interesting and recent finding was the effect of DPP4i on endothelial progenitor cells (EPCs). Circulating EPCs, originating from the bone marrow, have the ability to differentiate into endothelial cells, and by that play a key role in vasculogenesis [23]. In the cardiovascular system, the amount of circulating EPCs predicts the occurrence of cardiovascular events and death from cardiovascular causes [24]. Fadini et al. showed recently that DPP4i enhances the level of circulating EPCs [25].

Our study showed a beneficial association between pre-treatment with DPP4i and short-term cardiovascular outcomes among patients admitted with acute coronary syndrome. Alongside the history of CHF and age, chronic treatment with Sitagliptin, the only DPP4i given in our study population, was associated with a better KILLIP class on admission and reduced rates of 30-day MACE. Similar results were reported recently by Engel et al., which showed reduced incidence of CVD endpoints among diabetes patients treated with Sitagliptin compared to ones treated with sulphonyl-urea [26]. Moreoever, we documented additional lower rates of in-hospital complications (i.e. acute renal failure and pulmonary edema) with DPP4i treatment. This can be a random finding due to the small number of events, but it may also represent possible additional effects of DPP4 inhibition, such as the effect on endothelial dysfunction and inflammation [27].

It shoud be noted that Sitagliptin was the only DPP4 inhibitor that was presscribed in our dataset. Despite the similar mechanism of action, DPP4 inhibitors are not necessarily identical, and have pharmaco-dynamic and pharmaco-kinetic properties [28]. This was also shown to have clinical ramifications in a recent study, which showed that the mean amplitude of glycemic excursions, highest blood glucose level after supper, and hyperglycemia after breakfast were significantly lower with vildagliptin BID compared to once daily sitagliptin [29]. This question should be answered with randomized controlled trials that would be published in the near future.

### Study limitations

As all retrospective analysis, one cannot conclude a direct effect of DPP4i on cardiovascular events, and only the on-going randomized studies could give a direct answer to this question. The small sample size in the DPP4i group and the fact that treatment duration was not recorded may increase the chances that the findings are accidental and could be randome. the In addition, our survey was not designed to be a study for diabetics, and therefore the status of the disease, the rate of control as well as the duration are not available.

In conclusion, our data from ACSIS 2010 suggest that pre-treatment with DPP4i may be associated with a lower risk of in hospital complications and 30-day MACE among patients presenting with acute coronary syndrome. These findings suggest a possible role for DPP4i in appropriately selected high-risk patients with diabetes mellitus.

## Abbreviations

ACS: Acute Coronary Syndrome; ACSIS: Acute Coronary Syndrome Israeli Survery; CHF: Congestive Heart Failure; CI: Confidence Interval; DPPi: Di Pepidyl Peptidase 4 inhibitors; EPCs: Endothelial Progenitor Cells; GLP-1: Glucose Like Peptide-1; MACE: Major Adverse Cardiovascular Events; OR: Odds Ratio; STEMI: ST-Elevation Myocardial Infarction; UAP: Unstable Angina Pectoris.

## Competing interests

The authors declare that there are no competing interests.

## Authors’ contributions

EL, SG, IG and DG designed the study. SG, IG, NGY and SM designed and performed the statistical analysis. EL, SG, IG and DG drafted and reviewed the manuscript. EL, SG and DG gave final approval of the manuscript. All authors read and approved the final manuscript.
